# Are Non-Native Plants Perceived to Be More Risky? Factors Influencing Horticulturists' Risk Perceptions of Ornamental Plant Species

**DOI:** 10.1371/journal.pone.0102121

**Published:** 2014-07-08

**Authors:** Franziska Humair, Christoph Kueffer, Michael Siegrist

**Affiliations:** 1 Institute for Environmental Decisions – Consumer Behavior, ETH Zurich (Swiss Federal Institute of Technology), Zurich, Switzerland; 2 Institute of Integrative Biology, ETH Zurich (Swiss Federal Institute of Technology), Zurich, Switzerland; University of Essex, United Kingdom

## Abstract

Horticultural trade is recognized as an important vector in promoting the introduction and dispersal of harmful non-native plant species. Understanding horticulturists' perceptions of biotic invasions is therefore important for effective species risk management. We conducted a large-scale survey among horticulturists in Switzerland (*N* = 625) to reveal horticulturists' risk and benefit perceptions from ornamental plant species, their attitudes towards the regulation of non-native species, as well as the factors decisive for environmental risk perceptions and horticulturists' willingness to engage in risk mitigation behavior. Our results suggest that perceived familiarity with a plant species had a mitigating effect on risk perceptions, while perceptions of risk increased if a species was perceived to be non-native. However, perceptions of the non-native origin of ornamental plant species were often not congruent with scientific classifications. Horticulturists displayed positive attitudes towards mandatory trade regulations, particularly towards those targeted against known invasive species. Participants also expressed their willingness to engage in risk mitigation behavior. Yet, positive effects of risk perceptions on the willingness to engage in risk mitigation behavior were counteracted by perceptions of benefits from selling non-native ornamental species. Our results indicate that the prevalent practice in risk communication to emphasize the non-native origin of invasive species can be ineffective, especially in the case of species of high importance to local industries and people. This is because familiarity with these plants can reduce risk perceptions and be in conflict with scientific concepts of non-nativeness. In these cases, it might be more effective to focus communication on well-documented environmental impacts of harmful species.

## Introduction

Increasing international trade is of major concern in ecology and in environmental management because it enables species dispersal across biogeographic barriers [1]–[3]. At a global level, frequent and widespread introductions of non-native species have resulted in novel new species compositions and interactions in ecosystems [4]. In the literature it is generally assumed that new species can pose a major risk to biodiversity, ecosystem functioning, or human well-being [5], [6]. This is for example the case if introduced plant species (i.e., non-native plant species that arrived in Europe after the year 1500 A.D. thanks to human assistance) become dominant and possibly alter ecosystem functions, or when non-native species are associated with harm to other species or to the economy [7]. New and harmful species – i.e., invasive non-native species – are considered to be a leading cause of biodiversity loss [5].

Research and species management are increasingly concerned with drivers and pathways of species introductions and establishment, and the horticultural industry has been identified as a particularly important vector for the global dispersal of plant species (e.g., [2], [8], [9]). In order to design effective risk management strategies, it is therefore important to better understand what drives horticulturists' risk perceptions and what factors influence their willingness to engage in risk mitigation behavior. In this study, we therefore investigated the influence of the perceived non-nativeness of and the perceived familiarity (using the perceived horticultural importance as a proxy) with an ornamental plant species on horticulturists' perceptions of environmental risks. Further, we were also interested in the roles of risk and benefit perceptions related to risk mitigation behaviors.

In its function as a gatekeeper in plant dispersal, the horticultural industry has come into the spotlight of ecology and conservation management (e.g., [10]–[17]) because it has been recognized as an important driver of plant invasions. But horticulture is also an important partner in risk mitigation measures such as risk assessments (e.g., [18]) or consumer information, e.g., by directing consumers towards plants with a low environmental risk [19], informing about particular aspects of invasive species [20], or instructing consumers to appropriately handle risk species [21].

Thus, increasingly researchers, policy makers, and practitioners are interested in horticulturists' attitudes towards the invasion issue and in their incentives to engage in risk mitigation behavior. Burt *et al.* [22] found that awareness of the invasive plant problem and concern for the environment were important factors for horticulturists' willingness to engage in voluntary action. Another study conducted in the U.S. found that horticulturists believe that their risk mitigation behavior increases the reputation of horticulture in the public as an environmentally friendly industry [23]. Peters *et al.* [24] and Coats *et al.* [19] link horticulturists' interest in voluntary customer information to the perceived responsibility of the horticultural industry for the cost of damage resulting from non-native invasive ornamentals sold in their businesses. These studies described horticulturists' attitudes, but the psychological determinants that may shape risk perceptions towards non-native plant species were not examined.

For the successful integration of stakeholders into risk management strategies or to design adequate strategies to communicate environmental risks, understanding stakeholders' concerns and their underlying factors is essential [25], [26]. However, only little research has been conducted on what shapes risk perceptions in the context of biotic invasions (but see [27]). Starfinger *et al.* [28] and Stromberg *et al.* [29] report that risk perceptions towards non-native plant species have changed over time depending on varying economic needs and values held by different stakeholder groups. Fischer and van der Wal [30] examined attitudes by the public towards the management of a non-native invasive plant species (*Lavatera arborea*) and found that risk perceptions and support of management measures were connected to environmental values such as ecological balance in nature or naturalness (defined as nature untouched by humans). While those participants that were concerned about the loss of an ecological balance in nature stated a need for action, those who were concerned about naturalness preferred no intervention. In their qualitative studies among lay people, conservation volunteers, and ecologists, Selge and Fischer [31] and Selge *et al.* [32] found that the perceived harmfulness of a species was not necessarily linked to its origin (native vs. non-native). Instead, perceptions of invasion risks from certain species were rather shaped by the notions of negative impacts on nature, economic costs (e.g., for agriculture), available management options, or perceived attractiveness of the invasive species. To some participants it was important to acknowledge the human role in species dispersal and biotic invasions: Natural spread of species was of lesser concern than human-induced dispersal.

Risk research has shown that lay people rely on qualitative risk characteristics for assessing the risk associated with a hazard [25], [33]. Lay people use the familiarity with a hazard, the controllability of a hazard, the voluntariness to be exposed to it, its dreadfulness, or the severity of consequences to be expected as indicators for the risk. In the psychometric paradigm these qualitative aspects of a hazard could be reduced to the two dimensions *Dread Risk* and *Unknown Risk* [25]. The location of the hazard on these two dimensions largely explains why different hazards are seen differently: The more dread is perceived from a hazard and the more unfamiliar a hazard is, the more risk is attributed to the hazard in question. However the method used by Fischhoff *et al.* [33] is based on aggregated data and does not allow to make any statements about individual differences in risk perceptions [34].

People differ in their risk perceptions. Some people perceive a lot of risks associated with a specific hazard, whereas other people may not perceive any risks at all. The affect heuristic is one of the mechanisms that has been proposed for explaining individual differences in risk perceptions [35]. It is assumed that the more negative associations a hazard evokes in an individual, the more risky this person perceives the hazard. Familiarity with a hazard may result in a change of affect, i.e., less negative or more positive feelings associated with the hazard. It has been shown that repeated exposure is sufficient to create a positive evaluation of a stimulus [36]. Also, it has been found that familiarity with a hazard may positively contribute to its acceptance; this is when people become knowledgeable about a hazard because it is encountered on a regular basis, such as smoking or driving cars [37]. The mitigating effect of familiarity with a hazard on individual risk perceptions has been discussed in various contexts, e.g., concerning nuclear waste disposal where familiarity with the nuclear industry raises public acceptance of repositories [38]; in the context of investment decisions where investors prefer familiar financial products that are perceived to be easier to understand and less risky [39]; or, regarding natural hazards where people familiar with flooding (i.e., those living in floodplains) feel less threatened by flooding events compared to people inexperienced with floods [40].

The aim of our study was to examine determinants of horticulturists' perceptions of risks from plant invasions as well as factors influencing horticulturists' willingness to engage in risk mitigation behavior. For this purpose, a written survey was conducted among members of the Swiss Association of Horticulture (JardinSuisse), the largest horticultural association in Switzerland. Besides negative impacts of a species, risk communication on biotic invasions often also emphasizes the non-native origin of the harmful species in question. We therefore hypothesized that (i) perceived non-nativeness of a species will result in a higher risk perception of this plant. Based on previous findings, we further assumed that (ii) horticulturists' familiarity with a plant species reduces the perceived risk associated with this plant. A second set of aims for the present research was to examine (iii) horticulturists' attitudes towards regulation and voluntary actions to mitigate invasion risks. We expected that (iv) perceived risk of non-native plants has a positive and perceived benefit a negative influence on respondents' willingness to support risk management action against non-native invasive plant species.

## Methods

### Ethics Statement

This study was based upon written questionnaires with members of the Swiss Association of Horticulture. Participants were ensured that the data would be anonymized. According to the directives of ETH Zurich, opinion surveys do not need to be approved by the ethics commission.

### Procedure and Participants

In fall 2012, a postal questionnaire was sent out to 1331 businesses associated with the Swiss Association of Horticulture (JardinSuisse), and operating in German-speaking parts of Switzerland. Mailing addresses had been provided by JardinSuisse. For each company, the person mainly responsible for the plant assortment was asked to complete the questionnaire. The survey was accompanied by a letter and a prepaid return envelope. Participants were informed that the study aimed to investigate how non-native plants were perceived in Switzerland and what significance they had for horticulture. One month after the questionnaire had been mailed, a reminder was sent out to non-responders enclosing another copy of the questionnaire. Data collection lasted in total for two months.

### Questionnaire

In the first part of the questionnaire, our study participants were asked to make four judgments about 18 ornamental plant species (see paragraph below and Table 1). We asked the participants to i. rate the importance of each species for landscape design in Switzerland, ii. classify the origin of each species as native or non-native, iii. express the perceived environmental threat of each plant species (threat/no threat), and iv. rate on a six-point scale the importance of each species for their own business (see also the copy of the questionnaire, Questionnaire S1). The plant species appeared in a random order, however, all participants received the same questionnaire.

**Table 1:** Description of the ornamental plant species used in this study.

	Plant	Common English Name	Assessed Impact
***Status of Origin: Non-Native***			
1	*Buddleja davidii*	Butterfly bush	Cause damage
2	*Lonicera japonica*	Japanese honeysuckle	Cause damage
3	*Prunus laurocerasus*	Cherry laurel	Cause damage
4	*Robinia pseudoacacia*	Black locust	Cause damage
5	*Cornus sericea*	Red-osier dogwood	Potential to cause damage
6	*Lupinus polyphyllus*	Russell lupin	Potential to cause damage
7	*Viburnum rhytidophyllum*	Leatherleaf viburnum	Potential to cause damage
8	*Mahonia aquifolium*	Oregon grape	Potential to cause damage
9	*Paulownia tomentosa*	Princess tree	Potential to cause damage
10	*Sedum spurium*	Creeping sedum	Potential to cause damage
11	*Trachycarpus fortunei*	Chinese windmill palm	Potential to cause damage
12	*Lonicera henryi*	Henry's honeysuckle	Potential to cause damage
13	*Fallopia baldschuanica* *	Russian vine	None
14	*Syringa sp.*	Lilac	None
15	*Wisteria sp.*	Wisteria	None
***Status of Origin: Native***			
16	*Ilex aquifolium*	English holly	None
17	*Prunus spinosa*	Blackthorn	None
18	*Euonymus europaeus*	European spindle tree	None

Species are ordered according to their assessed impact. In the questionnaire, these plant species appeared in a random order.

*Note: S*tatus of origin for all species is taken from Flora indicativa [42].

Assessed impact is based on whether the species is listed on the Black-List, or on the Watch-List [43].

*At the time of our study, the use of *F. baldschuanica* in horticulture was allowed by Swiss authorities, although the species belongs to the *Fallopia*-complex, that was included in the list of prohibited plant species [46], but not in the Black- or the Watch-List.

A second set of items was used to explore risk and benefit perceptions from ornamental plant species. Further, we examined participants' attitudes towards risk mitigation behavior aimed at reducing the invasion risk of non-native ornamental plant species (e.g., increasing prices for species known to be invasive in Switzerland). Participants' general perception of risks emerging from non-native invasive plant species in Switzerland was measured on a 6-point scale, (Table 2, item 1). Perceived economic and cultural benefits of non-native plant species for the horticultural business in Switzerland were measured using eight items, e.g., “For my business, non-native plants are economically important”, (Table 2, items 2—9). Participants' attitudes towards risk mitigation behavior were examined using four items related to regulations of import and trade of non-native plants, (Table 2, items 10—13), and five items measuring the willingness to voluntarily promote the sale of native plant species (Table 2, items 14—18).

**Table 2:** Perceptions of risk and benefit, and attitudes towards the willingness to engage in risk mitigation behavior and factor loadings from principal component analysis.

Scales and Items		*M*(*SD*)	Factor loadings
***General Risk Perception***			
1	What is your general estimate of the size of the problem caused by invasive non-native plants in Switzerland?	3.99 (1.34)	
***Benefits*** **; Cronbach's ** ***α*** ** (scale mean): 0.84 (3.74)**			
2	For my business, non-native plants are economically important.	3.70 (1.74)	
3	It is easier to sell exotic plants than native species.	3.51 (1.53)	
4	The choice of plants has to be complemented by non-native plants because native ones do not possess all of the properties desired by the customers.	4.77 (1.43)	
5	New species and cultivars may improve biodiversity.	3.40 (1.60)	
6	If I don't sell a non-native plant because of the risk it poses to the environment, my customers may buy it in another store.	3.66 (1.75)	
7	To me, it is important to frequently offer my customers new species and cultivars.	3.79 (1.60)	
8	The cultural value of a horticultural landscape is essentially dependent on new species and cultivars.	2.97 (1.54)	
9	Non-native plants belong to our gardening culture.	4.12 (1.59)	
***Willingness to Engage in Risk Mitigation Behavior*** **; Cronbach's ** ***α*** ** (scale mean): 0.80 (4.08)**			
10	As long as one cannot rule out the possibility that a non-native plant will become invasive in Switzerland, a ban on the import of this plant should be imposed.	3.36 (1.83)	0.72
11*	In Switzerland, trade of non-native plants does not require legal regulations.	2.96 (1.85)	0.64
12*	There should be no restrictions on importation of non-native plants into Switzerland, as long as there is no evidence that these plants potentially become invasive in Switzerland.	3.70 (1.82)	0.65
13	No plants should be allowed for import into Switzerland that have been shown to have been invasive in another country.	4.50 (1.68)	0.57
14	Increase prices for plants that are included in the Black list (i.e. plants that had been listed by the Swiss Commission of Wild Plant Conservation to be invasive non-native plants in Switzerland, and that cause negative impacts in the context of biodiversity, health, and/or economy).	3.00 (1.93)	0.48
15	Remove plants from my stock that are listed on the Black-list.	4.64 (1.56)	0.68
16	Ban the sale of any non-native plant, until it has been shown that it does not pose a danger to humans or the environment.	3.90 (1.73)	0.76
17	Inform customers about invasive non-native plant species.	5.31 (1.03)	0.62
18	Promote the sale of native plants.	4.73 (1.40)	0.52

*Note*: Numbers vary from 608 to 622 because of missing data. Rating scales for item 1 went from 1 = ‘very small problem’ to 6 = ‘very big problem’, for items 2–13 from 1 = ‘not agree at all’ to 6 = ‘totally agree’, and for items 14–18 from 1 = ‘I cannot imagine at all’ to ‘I can imagine very well’.

*Item was recoded prior to principal component analysis. Some of the survey questions were adapted from Peters *et al.* [24] and Coats *et al.* [19].

In the last part of the questionnaire, participants were asked socio-demographic questions about their age, gender, educational level, business sector, main source of income, average number of full-time employees, and their function in the company (see also Table 3 and Questionnaire S1). Anonymized data from this survey will be provided upon request by the first author.

**Table 3:** Socio-demographic characteristics of the analysis sample (*N* = 625).

Variable	N	%
***Gender***		
Male	576	93
Female	41	7
***Educational Level***		
Primary/lower secondary school	22	4
Upper secondary vocational school/upper secondary university preparation school	297	49
College/university	174	28
Special training (e.g., gardener in chief)	119	19
***Business sector***		
Private consumer business	395	66
Wholesale market	30	5
Mixed clientele	172	29
***Main Source of Income***		
Horticulture, landscape architecture & gardening	219	36
Horticulture & landscape architecture	133	22
Gardening	86	14
Potted plants and cut flowers	77	13
Tree nurseries	43	7
Various combinations of horticultural sectors	47	8
***Average number of full-time employees per year***		
1–5	280	45
6–15	194	31
16–30	79	13
>30	65	11
**Function in the company**		
General manager/brand manager	538	89
Head of department	39	6
Other position, e.g., in administration	31	5
***Age (range 23–96 years)***	**M**	**SD**
In years	47	11

### Plants selected for this study

The list of plant species used in this study was compiled from a catalogue of ornamental plant species available in Switzerland provided by the Swiss Association of Horticulture [41]. We used this catalogue because we assumed that most participants would be familiar with these species. Species were chosen based on their status of origin in Switzerland [42], as well as based on plant risk assessments supervised by Swiss authorities (Black-list and Watch-list) [43]. Because we were particularly interested in perceptions related to non-native plant species – notably those with known or presumed negative impacts on biodiversity, health, and/or the economy – our selection consisted primarily of species from the Black-list (invasive, 4 species) and the Watch-list (potentially invasive, 8 species). Three non-native species were chosen that at the time of the study were not considered to pose any threat to the environment. Hence, our choice of species reflected a range of environmental risks from very invasive to non-invasive. Also, the 3 native species used in this study were not regarded to be environmentally risky by Swiss authorities and served as a control, see Table 1.

All of the species chosen were part of the basic training for prospective gardeners in Switzerland supported by JardinSuisse [44]. Further, these species were particularly promoted by the industry as ornamentals, or in the case of the spiny shrub *Prunus spinosa* as a natural protection of economically important plants against game browsing. Further, the native species *Ilex aquifolium* had been proposed by JardinSuisse as an alternative to *Buxus* species that in Switzerland are threatened by the non-native invasive box tree moth (*Cydalima perspectalis*).

In Switzerland, over 600 non-native plant species have been recorded (c. 20% of the total flora in Switzerland, [45]). Thereof, around 10% are considered to have negative effects, see also Info Flora [43] or (Annex 2; SFC, [46]). Thus, non-native and in particular, non-native invasive species used in this study are overrepresented compared to the actual situation in Switzerland.

### Statistical Analysis

Fisher's exact tests were performed using R version 2.15.2 (http://www.r-project.org), all other statistical analyses were performed using SPSS Version 20 (SPSS Inc., Chicago, IL, USA).

We applied a multiple linear regression analysis using the forced entry method to evaluate to what extent perceived risk and perceived benefits, as well as socio-demographic variables influenced horticulturists' willingness to engage in risk mitigation behavior. Socio-demographic variables were recoded into dummy variables: Gender classified as male = 0, female = 1; Age, categorized into three age classes of 21—40, 41—60 (reference category), and over 60 years at survey; Position in Business, classified as general manager or branch manager, or other (including heads of department, case handlers, trainees, or administrative stuff); Main Source of Income, with six categories: 1 = horticulture, landscape architecture, and gardening (reference category), 2 = horticulture and landscape architecture, 3 = gardening, 4 = potted plants and cut flowers, 5 = tree nurseries, and 6 = others (including different combinations of Categories 1—5 or supply services); Business Sector, categorized as wholesale market, private consumer business (reference category), or mixed clientele; Number of Full Time Employees, classified according to the Swiss horticultural market structure into the categories 1—5 (reference category), 6—15, 16—30, and over 30 employees.

## Results

### Respondents

The questionnaire was sent to 1331 members of the Swiss Association of Horticulture (JardinSuisse) in the German-speaking parts of Switzerland. The response rate was 47% (*N* = 625). Most respondents were male. This might reflect a general gender bias in the industry. The demographic characteristics of the study participants are described in Table 3.

### Associations between Perceived Origin and Perceived Environmental Threat

**Figure 1:**
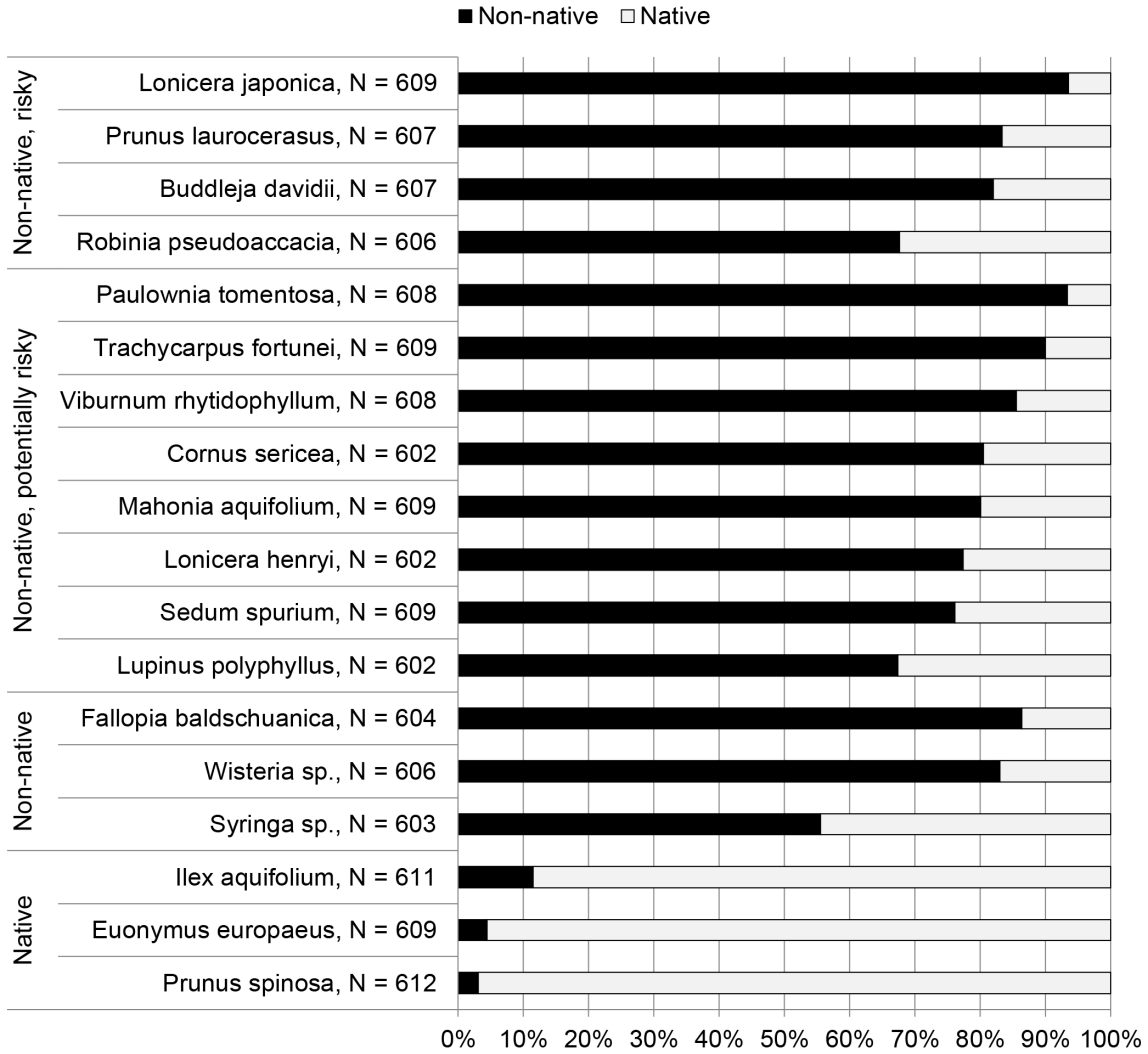
Perceived origin of plants used in this study.

Participants were presented with a list of 18 ornamental plant species and were asked to classify these plants as native or non-native. Irrespective of their true origin, all plants were classified as non-native by some and as native by other participants. The fraction among participants not following the classification of origin given in the literature accounted for between 6% and 44% (*M* = 20%) in the case of non-native species, and between 3% and 12% (*M* = 6%) in the case of native species (Figure 1).

**Figure 2:**
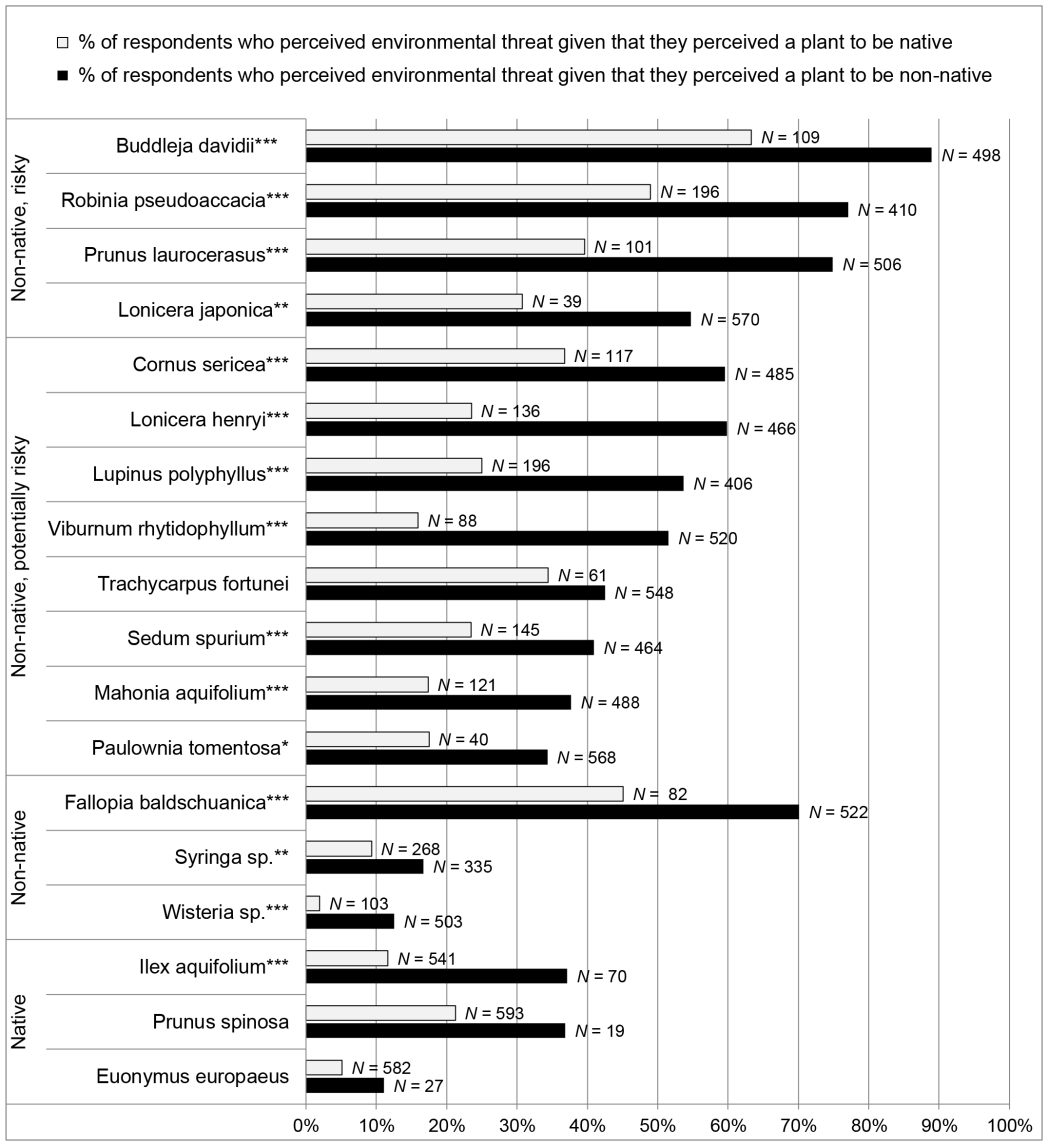
Association of Origin and Risk Perception. The percentages of participants who perceived a plant as an environmental threat depending on the perceived origin of the plant. *Note:* *p<.05, **p<.01, ***p<.001, Fisher's exact test, one-tailed. *N* corresponds to the number of study participants who classified a plant as either non-native or native.

In order to analyze the relationship between perceived origin and perceived environmental threat, for each plant we divided the participants into two groups, depending on whether they classified a given plant as native or as non-native. For both groups, we then calculated the percentage of participants that perceived the plant as an environmental threat (Table 4). We found for all plants a strong association between perceived origin and perceived environmental threat: independent of the true origin of the species, the fraction of participants who perceived a plant to be risky was larger among those that classified the plant as non-native than those that classified it as native (*p* = 0.05—0.001, Figure 2).

**Table 4:** Descriptives of variable perceived environmental threat if not handled correctly and point-biserial correlations between perceived importance and perceived environmental threat.

Plant name	% of Participants who Perceived Environmental Threat	Correlations: Importance Landscape Design in Switzerland vs. Environmental Threat	Correlations: Importance Own Business vs. Environmental Threat
*Buddleja davidii*	85	−0.35**	−0.26**
*Prunus laurocerasus*	70	−0.29**	−0.24**
*Robinia pseudoaccacia*	69	−0.28**	−0.28**
*Lonicera japonica*	54	−0.27**	−0.28**
*Sedum spurium*	37	−0.34**	−0.25**
*Virburnum rhytidophyllum*	46	−0.32**	−0.30**
*Lupinus polyphyllus*	45	−0.29**	−0.26**
*Paulownia tomentosa*	34	−0.28**	−0.21**
*Mahonia aquifolium*	33	−0.27**	−0.29**
*Lonicera henryi*	52	−0.26**	−0.27**
*Cornus sericea*	58	−0.24**	−0.15**
*Trachycarpus fortunei*	42	−0.22**	−0.18**
*Fallopia baldschuanica*	68	−0.35**	−0.36**
*Syringa sp.*	13	−0.16**	−0.17**
*Wisteria sp.*	11	−0.14**	−0.17**
*Ilex aquifolium*	15	−0.23**	−0.23**
*Prunus spinosa*	22	−0.19**	−0.22**
*Euonymus europaeus*	6	−0.11**	−0.16**

*Note*:

**p<.01;

Rating scales for perceived importance went from 1 = ‘absolutely unimportant’ to 6 = ‘very important’; perceived environmental threat: 0 = ‘no threat’ and 1 = ‘threat’; *N* varies between 569 and 610, reflecting missing values.

### Perceived Importance and Perceived Environmental Threat

The more important the plant was perceived to be for landscape design or for a participant's business, the less risky the plant was evaluated (Table 4). These correlations were rather small for the three native plants and two of the three non-native plants not considered to pose an environmental threat according to Swiss authorities.

### Risk and Benefit Perceptions

In average, our study participants rated the overall risk from non-native invasive plant species in Switzerland as medium (Table 2, item 1). We asked our study participants a set of questions referring to potential economic and cultural benefits generated by non-native plant species (Table 2, items 2—9). The internal reliability of the benefit scale was good (Cronbach's *α* = .84, *M* = 3.74, *SD* = .53). The large majority felt it was necessary to complement the product range of the horticultural industry with non-native plant species because native species were missing characteristics requested by customers. Although horticulturists tended to accept non-native plant species as a part of horticulture, only a minority felt that new species or varieties were essential for the cultural value of a garden. Participants diverged in their responses to three questions: whether non-native plant species were of economic importance for their businesses, whether non-native plants were easier to sell than native ones, and whether new species and varieties were a valuable addition to local biodiversity. In addition, horticulturists did not feel a particular need to regularly to present their customers with new species or cultivars, nor did they seem to fear business competition if they would cease to sell a non-native plant species judged harmful to the environment.

### Willingness to Engage in Risk Mitigation Behavior

To identify meaningful dimensions describing participants' willingness to engage in risk mitigation behavior, a principal component analysis (PCA) was performed on the items measuring attitudes towards import and trade regulations (Table 2, items 10—13), as well as on the items describing voluntary actions (Table 2, items 14—18). The first factor accounted for 40% of the total variance in the measures. The second factor however had an eigenvalue only marginally greater than one and it explained only 12% of the variance in the data. The scree-plot further suggested that a one-component solution should be favored. We therefore decided for a one-factor solution and the items described above were combined to a scale labeled *Willingness to Engage in Risk Mitigation Behavior*. The internal consistency of this scale was determined using Cronbach's alpha (*α* = .80, *M* = 4.08, response scales ranged from 1—6).

Most study participants supported an embargo on import of plant species that were invasive in another country, while only a minority was in favor of a regulatory-free trade of non-native plant species in Switzerland. However participants were rather reluctant to support preventive measures as long as the invasiveness of a plant was not demonstrated conclusively and the means of the ratings were slightly below and above the midpoint of the 6-point scale, respectively. We found particularly positive attitudes towards customer information, towards the promotion of native plant species, and towards the removal of problematic invasive plant species from the assortment. The options to renounce sale of any non-native plant species as a precautionary measure, or to increase prices for problematic non-native ornamentals were least welcomed.

### Multiple Linear Regression

We used linear regression analysis to evaluate to what extent perceived risk and perceived benefits, as well as socio-demographic variables – Gender, Age, Position in Business, Main Source of Income, Business Sector, and Number of Full Time Employees – influence Willingness to Engage in Risk Mitigation Behavior. Socio-demographic variables were recoded as dummy variables (Table 5).

**Table 5:** Results of multiple regression analysis with Willingness to Engage in Risk Mitigation Behavior as dependent variable.

Independent Variable	*B*	*SE*	β
**Socio-Demographic Variables**			
***Gender***	0.08	0.14	0.02
***Age***			
21–40 years	−0.01	0.08	0.00
41–60 years†	-	-	-
over 60 years	0.03	0.11	0.01
***Position in Business***	0.04	0.11	0.01
***Main Source of Income***			
Horticulture, landscape architecture, and gardening†			
Horticulture and landscape architecture	0.24	0.09	0.09**
Gardening	0.19	0.11	0.06
Potted plants and cut flowers	−0.12	0.11	−0.04
Tree nursery	−0.31	0.15	−0.08*
Others (combination of several sources of income)	−0.09	0.13	−0.02
***Business Sector***			
Wholesale market	−0.20	0.16	−0.04
Private consumer business†	-	-	-
Mixed clientele	0.04	0.08	0.02
***Number of Full Time Employees***			
1–5 employees†	-	-	-
6–15 employees	0.02	0.08	0.01
16–30 employees	−0.07	0.11	−0.02
Over 30 employees	−0.19	0.12	−0.06
*Psychological Variables*			
***Benefit***	−0.34	0.04	−0.35***
***Risk Perception***	0.30	0.03	0.39***

*Note*:

*p<0.05,

**p<0.01,

***p<0.001,

*N* = 541, *R^2^* = 0.48, adjusted *R^2^* = 0.46.

Dummy variable gender was coded as 0 = male, 1 = female. Position in Business was coded as 0 = other (e.g., head of department/administration), 1 = general manager or branch manager.

† Reference category.

In a multiple linear regression where all predictors were entered at once, our model explains 46% percent of the variance (Table 5). The variable describing general risk perception (β = 0.39, *p*<.001) and the benefit scale (β = −.35, *p*<.001) significantly influenced horticulturists' willingness to engage. Our study participants displayed high willingness to engage when risk perception related to non-native invasive plant species was high and when the perceived cultural or economic benefit emerging from non-native plants was perceived to be low. We also observed a weak dependence of the willingness to engage in risk mitigation behavior on the predictor variable Main Source of Income. However, only in two cases this weak dependency was statistically significant. All other socio-demographic variables did not significantly contribute to the participants' willingness to engage in risk mitigation.

In order to test how much variance was explained by the psychological variables Benefit and Risk Perception in addition to the socio-demographic variables, a hierarchical regression was conducted (Table S1). In the first step, socio-demographic variables were included (*R^2^* = 0.16). In the second step, we included psychological variables describing risk perceptions and perceptions of benefit. The additional inclusion of psychological variables caused *R^2^* to increase significantly by 32% (adjusted *R^2^* = 0.46). This analysis suggests that the psychological variables are more important than socio-demographic ones.

## Discussion

### General Discussion

Our data suggest that horticulturists' perceptions of environmental risks emerging from ornamental plant species were significantly influenced by the perceived origin (native vs. non-native) of the study species, and the perceived importance for landscape design in Switzerland and for participants' businesses. Horticulturists did not unanimously follow the classifications of a plant as native or non-native as given in the literature and used by Swiss authorities. Further many of our study participants accepted regulations of trade and declared a willingness to engage in various voluntary actions to mitigate invasion risks from non-native ornamentals. Thus, our data suggest that horticulturists seemed to be aware of the invasion issue and of the necessity to take preventive measures. Yet, participants perceived only medium environmental risks emerging from non-native invasive plant species in general. Voluntary actions to mitigate invasion risks from non-native ornamentals were significantly influenced by risk and benefit perceptions. Our data were derived from a large-scale survey among members of the Swiss Association of Horticulture that represents most horticulturists in Switzerland. To the best of our knowledge, no previous large-scale quantitative study has reported factors underlying horticulturists' perceptions of risk and attitudes towards the willingness to engage in risk mitigation behavior.

### Non-Nativeness as Risk Factor

The perceived origin of an ornamental plant species significantly influenced horticulturists' perceptions of environmental risks: Participants who perceived a plant species to be non-native were significantly more likely to perceive this species to be risky, compared to those participants who perceived the same species to be native. Participants might have perceived the non-nativeness of a species as a negative stereotype that characterizes non-native plant species in general as environmentally risky. Due to the correlational nature of our analysis, we can however not exclude the possibility that the perception of origin was influenced by the perceived riskiness of a plant species instead of the opposite.

Our finding that the perceived non-nativeness of a plant species might serve as a subjective risk metric among horticulturists sheds new light on the perceptions of risks among stakeholders in the management of plant species. Results from previous studies indicate that to non-academic stakeholders the origin of a species does not seem to be relevant for their risk perception; for them documented negative effects on the new environment matter [31], [32]. These results come from studies that elicited risk perceptions in focus groups and by using direct measures. This means that participants explicitly elaborated on the importance of the origin of a species for their perceptions of environmental risks. Using open group discussions is a particularly useful instrument to explore attitudes and beliefs towards an object in-depth instead of quantitatively analyzing attitude scores. However in some cases, participants in non-anonymous group discussions might be prompted to reason in a socially desirable way [47]. In the studies cited above, participants might have felt compelled to deny that their perceptions of risks were influenced by the non-nativeness of the species in question. In our study however, we indirectly measured associations of perceived origin and perceived environmental threat. In our questionnaire, we first asked the participants to classify a species as either native or non-native. Subsequently the participants were asked to evaluate the species' environmental threat. The ratings for these two separate questions were then used to calculate the impact of the perceived origin on perceived environmental threat.

The notion of a species' origin as subjective risk metric is particularly noteworthy given our finding that our participants did not unanimously follow the classification of origin used by Swiss authorities. Note that the latter finding might also (partially) reflect a bias caused by the unequal size of the groups of non-native (15 species), and native plant species (3 species). For example, if participants assumed that there was an equal probability for a species to be non-native or to be native, a tendency towards assessing non-native species as native might be expected in the case of such an unbalanced sample.

Given the importance of perceived origin for risk perception, diverging perceptions of which plant species is native and which is non-native might result in diverging risk perceptions and ultimately in diverging views on the necessity of management measures, such as, e.g., a precautionary ban on import for novel ornamental plant species.

### Familiarity or Benefit Mitigating Perceptions of Risk

Horticulturists' perceptions of environmental risk from a plant species were also significantly associated with perceptions of the importance of the species for landscape design in Switzerland and their businesses. This might be because either benefit from or familiarity with an ornamental plant species mitigated risk perceptions; both effects are well documented through risk perception studies performed in a wide range of contexts. On the one hand, there might be an inverse relationship between risk and benefit perceptions that was also shown in the contexts of technological risks, e.g., aviation, electric power, X-ray technology [33], [48]. Considering that horticulturists have more experiences with plant species that are important for their businesses or the industry, perceived importance might alternatively be interpreted as a proxy for horticulturists' familiarity with a species. This interpretation is supported by our findings that for most species there were positive associations of perceived native origin and perceived importance (*p*<0.01, |r| ∈ [0.15, 0.34]). Indeed, often it is not the native origin, but the novelty and the “exotic” origin of horticultural plants that are used as important selling arguments [15]. Familiarity might also explain why our participants tended to perceive non-native plants as native (Figure 1).

The apparent risk mitigation effect of familiarity we have observed is in line with previous research on the influence of perceived familiarity on risk perception from activities or technologies where it was found that perceived familiarity with a hazard significantly increased risk tolerance [33]. Such an effect of familiarity might be explained by an induced positive feeling through repeated exposure to the same stimulus (Mere Exposure Effect; [36]). Particularly when time and/or information are limited, affective evaluation influenced by positive feelings may then serve as a heuristic in judgments of risks or benefits and replace rational analysis of the available information In summary, our data suggest that work experience with an ornamental plant species affects horticulturists' risk awareness either through increased perceived benefit or a familiarity effect.

### Willingness to Engage in Risk Mitigation Behavior

Our results suggest that risk and benefit perceptions largely explain the variance in the reported willingness to engage in risk mitigation behavior. Both variables had a comparable degree of importance in the model, yet effects were opposite in direction. Horticulturists' willingness to engage was reduced if they perceived benefits from non-native plant species, i.e., economic or cultural values, or specific plant characteristics thought to be important to customers. General perceptions of risk from non-native invasive plant species seemed to increase horticulturists' reported willingness to engage.

We found particularly high approval of some voluntary actions; namely customer information, promotion of native plant species, and the removal of known harmful non-native plant species from the stock. Participants also agreed on regulations of trade of non-native plant species in general, albeit it seemed that voluntary actions were preferred over government-controlled regulations. Particularly unpopular was the option to voluntarily increase sales prices for non-native ornamentals known to be invasive. Equally in the studies of Gagliardi and Brand [23] and Barbier *et al.* [49] market-based management measures such as taxes or fees were the most undesired ones. Besides a general dislike for taxes or fees, Barbier *et al.* [49] also discuss horticulturists' concerns over scientific uncertainties that might make it difficult to design and implement adequate market-based measures.

In general, our results are in line with the findings of other authors, who reported horticulturists' expressed willingness to participate in risk mitigation processes, e.g., in a voluntary code of conduct [19], [23], [24], [49], [50]. In a study among horticulturists in the U.S., Burt *et al.* [22] found environmental concern, the cultivation of the reputation of an environmentally responsible business, and customer demand among the most influential incentives to engage in risk mitigation behavior.

Interestingly, our data indicate that among horticulturists there is no clear support for precautionary measures targeted at non-native species in general. In ecology and environmental management, it is widely assumed that precautionary measures are likely more effective than a later cure [6], [51]. However, whereas a ban on import for non-native ornamentals with known invasive potential was well received by our study participants, the precautionary ban on import or sale of non-native species despite missing knowledge about its invasive potential did not receive much support. Gagliardi and Brand [23] found ambiguous attitudes towards the ban on invasive ornamental plants among nursery and landscape industry members in the U.S. state Connecticut: Far more study participants opposed the ban on economically important than unimportant plant species. Thus, also among Swiss horticulturists, expected loss of economic benefits from the sale of non-native species might have fueled refusal of preventive measures.

### Limitations and Further Research

Some limitations of the presented results have to be discussed. We cannot exclude that estimations of risks and reported perceived importance of ornamental plant species might reflect a self-presentation bias. That is, some horticulturists might have had the tendency to present their businesses in the best light and thus to report lower perceptions of risk and of importance of a study plant than are actually experienced. Or, some horticulturists might have strategically underestimated plant risks in order to prevent further governmental regulations. Also, perceptions of risk from non-native invasive plant species in general were measured using only a single-item and therefore the reliability of this measure might be low. Usually, single-items also explain less variance. However, we found that general risk perceptions had a substantial influence on the regression model. Thus, using a single-item might have led to an underestimation of the influence of the general risk perceived.

In order to increase effectiveness in risk communication and to facilitate concerted risk management, future research may continue to reveal horticulturists' attitudes and perceptions towards non-native plant species and to examine further factors determining horticulturists' willingness to engage in risk mitigation behavior, or these factors framing subjective risk perceptions. Further, longitudinal studies among horticulturists would allow observing if their perceptions of environmental risks related to non-native invasive plant species change over time, what role experience with plant invasions may play in the formation of risk perceptions, and if risk communication and other management strategies are successful [52].

### Implications for Management

Our finding that the perceived non-nativeness of an ornamental plant species is associated with increased environmental risk perception seems to support the prevalent practice in risk communication that emphasizes the non-native origin of harmful species. However, according to our data such a communication strategy might bear the risk of being ineffective. Among our study participants, the perceptions of which ornamental plant species are native (or non-native) were often not congruent with the classifications proposed by experts in the literature and adopted by Swiss authorities. Even among academic experts there exists a high diversity of alternative concepts of the non-native origin of a plant species in Europe [53]. Such divergent perceptions of a key concept might hinder consensus building and therefore weaken risk communication. It might be possible to better explain to non-experts the scientific understanding of the non-native biogeographic origin of a species, but such clarification of the underlying science might not suffice. When a socially deeply rooted metaphor such as ‘non-native’ is employed in science communication, its understanding can be influenced by other legitimate factors such as – in this case – broader concepts of nativeness or familiarity [53]. Indeed, if our interpretation is correct (see above) the familiarity of horticulturists with a plant might be the reason why perceptions of environmental risks from particular ornamental plant species differed between horticulturists and academic experts or authorities; especially in the case of ornamental plants of high importance to the horticultural industry. Horticulturists' familiarity with a non-native species might lead to a cognitive conflict with the expert definition of the non-native origin of a species: a species that is characterized as ‘foreign’ by experts is ‘familiar’ to horticulturalists due to their daily work. Thus, while the non-native origin of a species can be an important scientific concept for understanding why a species is harmful, it can be problematic in risk communication, especially in the case of those species that are long-established and well-known in a country and therefore familiar to the public or particular stakeholder groups. As an alternative it might in these cases be more effective to focus communication on well-documented environmental impacts of harmful species (e.g., [30]). The communication choices that are most intuitive to involved scientists – for instance fear-based stereotypes in invasive species or in climate change communication – are not always the most effective ones [54].

## Supporting Information

Table S1 **Hierarchical regression analysis with Willingness to Engage in Risk Mitigation Behavior as dependent variable.**
(PDF)

Questionnaire S1 **Written questionnaire with members of the Swiss Association of Horticulture, 2012.**
(PDF)
