# Supplementary material for: Are Non-Native Plants Perceived to Be More Risky? Factors Influencing Horticulturists' Risk Perceptions of Ornamental Plant Species
Source: PLoS One. 2014 Jul 8;9(7):e102121. doi: 10.1371/journal.pone.0102121 (PMC4086969; doi:10.1371/journal.pone.0102121)
Supplement: Table S1 — Hierarchical regression analysis with Willingness to Engage in Risk Mitigation Behavior as dependent variable. (PDF) [file pone.0102121.s001.pdf]

**Table S1. Hierarchical regression analysis with Willingness to Engage in Risk Mitigation Behavior as dependent variable.** In the first model including socio-demographic variables only, the model explained 16% of the variance, $F(14, 526) = 7.03$, $p < 0.001$. Four variables denoting the main source of income (*Horticulture and landscape architecture* (β = 0.10), *Potted plants and cut flowers* (β = -0.10), *Tree nursery* (β = -0.23), and *Others (combination of several sources of income)* (β = -0.15)), as well as the variable *Over 30 employees* (β = -0.12) referring to the number of full time employees per year showed a significant influence on horticulturists willingness to act. The additional inclusion of the psychological variables describing risk perceptions (β = 0.39) and perceptions of benefit (β = -0.35) caused $R^2$ to increase significantly by 32% $F(2, 524) = 29.62$ , $p < 0.001$, (adjusted $R^2$ = 0.46). Only the contributions of the variables *Horticulture and landscape architecture* (β = 0.09) and *Tree nursery* (β = -0.08) remained significant, and particularly the proportion of variance accounted for *Tree nursery* decreased considerably.

| | Model 1 (a) | | | Model 1 (b) | | |
|---|---|---|---|---|---|---|
| **Independent Variable** | **B** | **SE** | **β** | **B** | **SE** | **β** |
| *Socio-Demographic Variables* | | | | | | |
| ***Gender*** | 0.215 | 0.174 | 0.051 | 0.075 | 0.138 | 0.018 |
| ***Age*** | | | | | | |
| 21 - 40 years | -0.08 | 0.095 | -0.035 | -0.008 | 0.075 | -0.004 |
| 41 - 60 years† | - | - | - | - | - | - |
| over 60 years | -0.066 | 0.134 | -0.021 | 0.026 | 0.106 | 0.008 |
| ***Position in Business*** | -0.146 | 0.136 | -0.046 | 0.036 | 0.108 | 0.011 |
| ***Main Source of Income*** | | | | | | |
| Horticulture, landscape architecture, and gardening† | - | - | - | - | - | - |
| Horticulture and landscape architecture | 0.25 | 0.113 | 0.10* | 0.237 | 0.089 | 0.09** |
| Gardening | 0.242 | 0.139 | 0.08 | 0.191 | 0.11 | 0.063 |
| Potted plants and cut flowers | -0.291 | 0.138 | -0.10* | -0.119 | 0.11 | -0.039 |
| Tree nursery | -0.932 | 0.183 | -0.23*** | -0.312 | 0.149 | -0.08* |
| Others (combination of several sources of income) | -0.572 | 0.166 | -0.15*** | -0.091 | 0.134 | -0.024 |
| ***Business Sector*** | | | | | | |
| Wholesale market | -0.313 | 0.206 | -0.066 | -0.201 | 0.163 | -0.042 |
| Private consumer business† | - | - | - | - | - | - |
| Mixed clientele | -0.089 | 0.1 | -0.039 | 0.036 | 0.08 | 0.016 |

| | | | | | | |
|---|---|---|---|---|---|---|
| ***Number of Full Time Employees*** | | | | | | |
| 1 - 5 employees† | - | - | - | - | - | - |
| 6 - 15 employees | 0.049 | 0.101 | 0.022 | 0.016 | 0.08 | 0.007 |
| 16 - 30 employees | -0.191 | 0.136 | -0.063 | -0.068 | 0.108 | -0.022 |
| over 30 employees | -0.397 | 0.154 | -0.12* | -0.192 | 0.123 | -0.056 |
| *Psychological Variables* | | | | | | |
| ***Benefit*** | | | | -0.336 | 0.035 | -0.35*** |
| ***Risk Perception*** | | | | 0.298 | 0.028 | 0.39*** |
| | | | | | | |
| **$R^2$** | | 0.16 | | | 0.46 | |
| **F** | | 7.03*** | | | 29.62*** | |
| **ΔF** | | N.A. | | | 158.353 | |

*Note*: *$p$ < 0.05, **$p$ < 0.01, ***$p$ < 0.001, $N$ = 541.

Dummy variable gender was coded as 0 = male, 1 = female.

Position in Business was coded as 0 = other (e.g., head of department / administration), 1 = general manager or branch manager.

†Reference category.
