# Supplementary material for: Are Non-Native Plants Perceived to Be More Risky? Factors Influencing Horticulturists' Risk Perceptions of Ornamental Plant Species
Source: PLoS One. 2014 Jul 8;9(7):e102121. doi: 10.1371/journal.pone.0102121 (PMC4086969; doi:10.1371/journal.pone.0102121)
Supplement: Questionnaire S1 — Written questionnaire with members of the Swiss Association of Horticulture, 2012. (PDF) [file pone.0102121.s002.pdf]

Questionnaire S1. Written questionnaire with members of the Swiss Association of Horticulture, 2012.

In our survey, we often use the term non-native plant. Please try to answer the following question as spontaneously as possible.

1. *For a moment, think about non-native plants. What are the first three words or images that spontaneously come to your mind?*

Word/image 1: ___________________________________

Word/image 2: ___________________________________

Word/image 3: ___________________________________

2. *You may now evaluate the words or images you just noted using a scale. Please indicate for every word or image what feeling you have.*

	very negative -3	-2	-1	0	1	2	very positive 3
Word/image 1:	☐	☐	☐	☐	☐	☐	☐
Word/image 2:	☐	☐	☐	☐	☐	☐	☐
Word/image 3:	☐	☐	☐	☐	☐	☐	☐

JardinSuisse has published the catalogue „Plants for our gardens". This catalogue also lists the plants that are listed in questions 1 – 4 of this paragraph.

1. *In your opinion, how important are the following plants for landscape design in Switzerland? Please check what best applies to you.*

	absolutely unimportant 1	2	3	4	5	very important 6
Wisteria (*Wisteria sp.*)	☐	☐	☐	☐	☐	☐
Oregon grape (*Mahonia aquifolium*)	☐	☐	☐	☐	☐	☐
Red-osier dogwood (*Cornus sericea*)	☐	☐	☐	☐	☐	☐
Cherry laurel (*Prunus laurocerasus*)	☐	☐	☐	☐	☐	☐
European spindle tree (*Euonymus europaeus*)	☐	☐	☐	☐	☐	☐
Henry's honeysuckle (*Lonicera henryi*)	☐	☐	☐	☐	☐	☐
Blackthorn (*Prunus spinosa*)	☐	☐	☐	☐	☐	☐
Russell lupin (*Lupinus polyphyllus*)	☐	☐	☐	☐	☐	☐
Creeping sedum (*Sedum spurium*)	☐	☐	☐	☐	☐	☐
Japanese honeysuckle (*Lonicera japonica*)	☐	☐	☐	☐	☐	☐
Princess tree (*Paulownia tomentosa*)	☐	☐	☐	☐	☐	☐
Lilac (*Syringa sp.*)	☐	☐	☐	☐	☐	☐
Black locust (*Robinia pseudoacacia*)	☐	☐	☐	☐	☐	☐
Russian vine (*Fallopia baldschuanica / Fallopia aubertii*)	☐	☐	☐	☐	☐	☐
English holly (*Ilex aquifolium*)	☐	☐	☐	☐	☐	☐
Butterfly bush (*Buddleja davidii*)	☐	☐	☐	☐	☐	☐
Leatherleaf viburnum (*Viburnum rhytidophyllum*)	☐	☐	☐	☐	☐	☐
Chinese windmill palm (*Trachycarpus fortunei*)	☐	☐	☐	☐	☐	☐

2. *In your opinion, do the plants listed below belong to the native flora or are they non-native?*

	native 1	non-native 2
Wisteria (*Wisteria sp.*)	☐	☐
Oregon grape (*Mahonia aquifolium*)	☐	☐
Red-osier dogwood (*Cornus sericea*)	☐	☐
Cherry laurel (*Prunus laurocerasus*)	☐	☐
European spindle tree (*Euonymus europaeus*)	☐	☐
Henry's honeysuckle (*Lonicera henryi*)	☐	☐
Blackthorn (*Prunus spinosa*)	☐	☐
Russell lupin (*Lupinus polyphyllus*)	☐	☐
Creeping sedum (*Sedum spurium*)	☐	☐
Japanese honeysuckle (*Lonicera japonica*)	☐	☐
Princess tree (*Paulownia tomentosa*)	☐	☐
Lilac (*Syringa sp.*)	☐	☐
Black locust (*Robinia pseudoacacia*)	☐	☐
Russian vine (*Fallopia baldschuanica / Fallopia aubertii*)	☐	☐
English holly (*Ilex aquifolium*)	☐	☐
Butterfly bush (*Buddleja davidii*)	☐	☐
Leatherleaf viburnum (*Viburnum rhytidophyllum*)	☐	☐
Chinese windmill palm (*Trachycarpus fortunei*)	☐	☐

3. *Is there any threat that the following plants spread and have negative effects on the environment if they are not handled correctly?*

	no threat<br>1	threat<br>2
Wisteria (*Wisteria sp.*)	☐	☐
Oregon grape (*Mahonia aquifolium*)	☐	☐
Red-osier dogwood (*Cornus sericea*)	☐	☐
Cherry laurel (*Prunus laurocerasus*)	☐	☐
European spindle tree (*Euonymus europaeus*)	☐	☐
Henry's honeysuckle (*Lonicera henryi*)	☐	☐
Blackthorn (*Prunus spinosa*)	☐	☐
Russell lupin (*Lupinus polyphyllus*)	☐	☐
Creeping sedum (*Sedum spurium*)	☐	☐
Japanese honeysuckle (*Lonicera japonica*)	☐	☐
Princess tree (*Paulownia tomentosa*)	☐	☐
Lilac (*Syringa sp.*)	☐	☐
Black locust (*Robinia pseudoacacia*)	☐	☐
Russian vine (*Fallopia baldschuanica / Fallopia aubertii*)	☐	☐
English holly (*Ilex aquifolium*)	☐	☐
Butterfly bush (*Buddleja davidii*)	☐	☐
Leatherleaf viburnum (*Viburnum rhytidophyllum*)	☐	☐
Chinese windmill palm (*Trachycarpus fortunei*)	☐	☐

4. *How important are the following plants for your own business?*

	absolutely unimportant<br>1	2	3	4	5	very important<br>6
Wisteria (*Wisteria sp.*)	☐	☐	☐	☐	☐	☐
Oregon grape (*Mahonia aquifolium*)	☐	☐	☐	☐	☐	☐
Red-osier dogwood (*Cornus sericea*)	☐	☐	☐	☐	☐	☐
Cherry laurel (*Prunus laurocerasus*)	☐	☐	☐	☐	☐	☐
European spindle tree (*Euonymus europaeus*)	☐	☐	☐	☐	☐	☐
Henry's honeysuckle (*Lonicera henryi*)	☐	☐	☐	☐	☐	☐
Blackthorn (*Prunus spinosa*)	☐	☐	☐	☐	☐	☐
Russell lupin (*Lupinus polyphyllus*)	☐	☐	☐	☐	☐	☐
Creeping sedum (*Sedum spurium*)	☐	☐	☐	☐	☐	☐
Japanese honeysuckle (*Lonicera japonica*)	☐	☐	☐	☐	☐	☐
Princess tree (*Paulownia tomentosa*)	☐	☐	☐	☐	☐	☐
Lilac (*Syringa sp.*)	☐	☐	☐	☐	☐	☐
Black locust (*Robinia pseudoacacia*)	☐	☐	☐	☐	☐	☐
Russian vine (*Fallopia baldschuanica / Fallopia aubertii*)	☐	☐	☐	☐	☐	☐
English holly (*Ilex aquifolium*)	☐	☐	☐	☐	☐	☐
Butterfly bush (*Buddleja davidii*)	☐	☐	☐	☐	☐	☐
Leatherleaf viburnum (*Viburnum rhytidophyllum*)	☐	☐	☐	☐	☐	☐
Chinese windmill palm (*Trachycarpus fortunei*)	☐	☐	☐	☐	☐	☐

In the following, we would like to ask you some general questions about so-called non-native plants.

By non-native plants, we mean plants that have been introduced to Switzerland by humans, deliberately or undeliberately, that originate from foreign regions, mostly different continents, and that are established here in the wild.

Definition according to the Swiss Commission of Wild Plant Conservation, since January 2012 Info Flora

1. *Many garden centers sell non-native plants. We are interested in why these plants are included in their offers. Please specify to what extent you agree with the following statements.*

		not agree at all 1	2	3	4	5	totally agree 6
a	For my business, non-native plants are economically important.	☐	☐	☐	☐	☐	☐
b	It is easier to sell exotic plants than native species.	☐	☐	☐	☐	☐	☐
c	The choice of plants has to be complemented by non-native plants because native ones do not possess all of the properties desired by the customers.	☐	☐	☐	☐	☐	☐
d	New species and cultivars may improve biodiversity.	☐	☐	☐	☐	☐	☐
e	If I do not sell a non-native plant because of the risk it poses to the environment, my customers may buy it in another store.	☐	☐	☐	☐	☐	☐
f	To me, it is important to regularly offer my customers new species and cultivars.	☐	☐	☐	☐	☐	☐
g	The cultural value of a horticultural landscape is essentially dependent on new species and cultivars.	☐	☐	☐	☐	☐	☐
h	Non-native plants cope particularly well with environmental change.	☐	☐	☐	☐	☐	☐
i	Non-native plants belong to our gardening culture.	☐	☐	☐	☐	☐	☐

In the following paragraph, we will ask you questions about so-called invasive non-native plants.

By non-native plants, we mean plants that have been introduced to Switzerland by humans, deliberately or undeliberately, that originate from foreign regions, mostly different continents, and that are established here in the wild. Non-native plants are called invasive if it is known or if it has to be assumed that they spread at uncontrollable rates and that they produce such a high stand density that biodiversity or its sustainable use may be compromised, or that humans, animals, or the environment may be threatened.

Definition according to the Release Ordinance (SR 814.911 Art. 3f) and to the Swiss Commission of Wild Plant Conservation, since January 2012 Info Flora

1. *Many garden centers offer non-native plants that may become invasive if not handled correctly, that is, they may have negative impacts on humans, animals, or the environment. We would like to learn more why these plants are sold in Switzerland. Please check what best applies to you.*

		not agree at all<br>1	2	3	4	5	totally agree<br>6
a	It is hard to remove some invasive non-native plants because there are no appropriate alternative plant species.	☐	☐	☐	☐	☐	☐
b	If I know that a non-native plant is a problem somewhere, I do not sell it.	☐	☐	☐	☐	☐	☐
c	Invasive plants may have characteristics such as fast growth or easy propagation that make them an interesting garden plant.	☐	☐	☐	☐	☐	☐

2. *What is your general estimate of the size of the problem caused by invasive non-native plants in Switzerland?*

very small problem<br>1	2	3	4	5	very big problem<br>6
☐	☐	☐	☐	☐	☐

The following part of our survey refers to the legal regulations that should be enacted by Switzerland to regulate import, trade, and the proper handling of non-native plants.

In your opinion, how should import and trade of non-native species be regulated? Please indicate how much you agree with the following statements.

		not agree at all 1	2	3	4	5	totally agree 6
a	As long as one cannot rule out the possibility that a non-native plant will become invasive in Switzerland, a ban on the import of this plant should be imposed.	☐	☐	☐	☐	☐	☐
b	In Switzerland, trade of non-native plants does not require legal regulations.	☐	☐	☐	☐	☐	☐
c	There should be no restrictions on importation of non-native plants into Switzerland, as long as there is no evidence that these plants potentially become invasive in Switzerland.	☐	☐	☐	☐	☐	☐
d	No plants should be allowed for import into Switzerland that have been shown to have been invasive in another country.	☐	☐	☐	☐	☐	☐

The green industry is required to inform its customers about characteristics of plants and their effects on organisms and the environment. Further, the green industry has to instruct its customers about the appropriate handling of plants (e.g., proper disposal). These are the requirements of the Federal Act on the Protection of the Environment as well as the Release Ordinance (Duty to Inform Regulation).

How much do you agree with the following statements regarding the Duty to Inform Regulation? Please check what best applies to you.

		do not agree 1	2	3	4	5	agree 6
a	The Duty to Inform regulation will lead to a loss of sales of economically important plants.	☐	☐	☐	☐	☐	☐
b	The Duty to Inform regulation will have a positive influence on the public image of the horticultural industry.	☐	☐	☐	☐	☐	☐
c	The Duty to Inform regulation will result in an increasing number of banned species that are not allowed to be sold.	☐	☐	☐	☐	☐	☐
d	The Duty to Inform regulation will result in a replacement of the sale of alien plants by the sale of plants that are considered unproblematic by legislature (alternative plants).	☐	☐	☐	☐	☐	☐
e	The Duty to Inform regulation is reasonable for a (not further specified) selection of plants.	☐	☐	☐	☐	☐	☐
f	The Duty to Inform regulation is practical for a (not further specified) selection of plants.	☐	☐	☐	☐	☐	☐

We now propose a number of concrete measures in the handling of non-native plants. These measures go beyond the legal regulations.

We are interested to learn if you can imagine voluntarily participating in one or in several of the following measures. Please check what best applies to you.

		I cannot imagine at all 1	2	3	4	5	I can imagine very well 6
a	Increase prices for plants that are included in the Black list (i.e. plants that had been listed by the Swiss Commission of Wild Plant Conservation to be invasive non-native plants in Switzerland, and that cause negative impacts in the context of biodiversity, health, and/or economy)	☐	☐	☐	☐	☐	☐
b	Remove plants from my stock that are listed on the Black-list.	☐	☐	☐	☐	☐	☐
c	Ban the sale of any non-native plant, until it has been shown that it does not pose a danger to humans or the environment.	☐	☐	☐	☐	☐	☐
d	Inform customers about invasive non-native plant species.	☐	☐	☐	☐	☐	☐
e	Promote the sale of native plants.	☐	☐	☐	☐	☐	☐

Different actors assess the risk whether a non-native plant will cause damage in Switzerland, or whether it has the potential to cause damage.

1. *Until to date, to what extent has the green industry been included in creating lists with invasive plants, with plants that have the potential to become invasive, or with plants that are (no longer) allowed to be traded?*

too little 1	accurately 2	excessively 3
☐	☐	☐

2. *In your opinion, what influence should the green industry have in future risk assessments of non-native plants compared to the prevalent practice?*

less 1	the same 2	more 3
☐	☐	☐

Finally, we ask you to provide some information about your business as well as about yourself.

1. In what business sector are you mainly working?

a	wholesale market	☐
b	private consumer business	☐
c	mixed clientele	☐

2. In what sector of the green industry are you mainly working? Please differentiate between the main source of income and the additional income.

		main source of income	additional income
a	horticulture / landscape architecture	☐	☐
b	landscaping / gardening	☐	☐
c	supply services	☐	☐
d	potted plants and cut flowers	☐	☐
e	tree nursery	☐	☐

3. What is the number of employees that on average have been working in your company for the past three years? (Counted up to full-time jobs during peak season)?

a	1 - 5 full-time jobs	☐
b	6 - 15 full-time jobs	☐
c	16 - 30 full-time jobs	☐
d	more than 30 full-time jobs	☐

4. What is your function in your company?

a	general management	☐
b	branch management	☐
c	head of department	☐
d	case handling	☐
e	training in the horticultural sector	☐
f	internship	☐
g	administration	☐
h	other..	

5. What is your gender?

a	male	☐
b	female	☐

6. What is your year of birth?

1	9		

7. What is currently your highest level of education?

a	primary school	☐
b	lower secondary school	☐
c	upper secondary vocational school	☐
d	upper secondary university preparation school	☐
e	college / university	☐
f	others...	
